# Effects of Caulis Spatholobi Polysaccharide on Immunity, Intestinal Mucosal Barrier Function, and Intestinal Microbiota in Cyclophosphamide-Induced Immunosuppressive Chickens

**DOI:** 10.3389/fvets.2022.833842

**Published:** 2022-03-18

**Authors:** Yao Cui, Wenjing Sun, Qinmei Li, Kaijun Wang, Yuhan Wang, Feifei Lv, Xiaogang Chen, Xiaomin Peng, Ying Wang, Jiang Li, Hongbin Si

**Affiliations:** State Key Laboratory for Conservation and Utilization of Subtropical Agro-Bioresources, College of Animal Science and Technology, Guangxi University, Nanning, China

**Keywords:** Caulis Spatholobi polysaccharide, immunosuppression, intestinal microflora, intestinal mucosal barrier, cyclophosphamide, immune enhancer

## Abstract

The protective effects of Caulis Spatholobi polysaccharide (CSP) on immune function, intestinal mucosal barrier, and intestinal microflora in cyclophosphamide (CY)-induced immunosuppressed chickens have been rarely reported. This study was designed to investigate the cecal microbiota in chickens and to elucidate the immune mechanism involved in the CSP effect on CY induced-immunosuppressed chickens. A total of 288 cocks were equally divided into six groups and used to evaluate the immune effect of CSP. Results showed that the CSP increased the body weight and immune organ index of immunosuppressed chickens, significantly increased the secretion of cytokines (IL-4, IL-10) and immunoglobulins (IgG, IgM) in sera of chickens, and restored the body immune function. The CSP reduced intestinal injury of the jejunum and ileum, increased the ratio of the intestinal villus height to crypt depth (V/C), improved the expression of tight junction protein, and protected intestinal health. The CSP activated the toll-like receptor (TLR)/MyD88/NF-κB pathway and enhanced the expression of TLR4, MyD88, NF-κB, Claudin1, and Zo-1, protecting the intestinal tract. High-throughput sequencing of the 16S rRNA gene showed that CSP increased species richness, restored CY-induced intestinal microbiome imbalance, and enhanced the abundance of *Lactobacillus* in the intestinal tract. In conclusion, our study provided a scientific basis for CSP as an immune enhancer to regulate intestinal microflora and protect intestinal mucosal damage in chickens.

## Introduction

Immunosuppression is a temporary or permanent dysfunction of the immune system (1, 2). Infection, stress, and abuse of antibiotics may cause immunosuppression in the body (3). Under immunosuppression, animals lose their ability to combat infections and viruses (4). For example, when chickens are immunosuppressed, they are more sensitive to pathogens like infectious bursal disease and *Escherichia coli* (5, 6). In this case, low antibody levels and failure to vaccinate will also lead to increased morbidity and mortality of chickens, bringing huge economic losses to the breeding industry (7). For example, manual error and drug abuse will also lead to vaccination failure, drug resistance, and other adverse consequences (8). Therefore, it is essential to develop safe and effective immune enhancers to combat immunosuppression in chickens.

A polysaccharide is a large polymeric sugar carbohydrate formed by glycosidic bonds (9). It has been reported that polysaccharides isolated from natural plants have various biological activities, such as antiviral, anti-inflammatory, antitumor, antioxidation, and immunoenhancing, and have low toxicity and slight side effects. Among them, immunoenhancing activities are the most significant, so they are generally used as immunomodulators (10, 11). For example, *Polygonatum sibiricum* polysaccharide (12), acid epimedium polysaccharide (8), Taishan Pinus massoniana pollen polysaccharide, and propolis (13) can promote immune response and regulate the immune state of chickens. These results strongly suggest that polysaccharides can be used as potent immunostimulants.

Cyclophosphamide (CY) is one of the most commonly used broad-spectrum anticancer drugs with salicylic acid, and it is also an immunosuppressant. In addition, CY disrupts the intestinal mucosal barrier and the gut microbiome (2, 14, 15). The intestinal tract is a major digestive and immune organ. The intestinal tract has a barrier function that can effectively prevent various parasitic bacteria and their toxins from migrating to the extra-intestinal tissues and organs, and prevents the body from being harmed by endogenous microorganisms and toxins (16). Under normal circumstances, there are plenty of bacteria in the intestinal tract. All kinds of bacteria interact and depend on each other, constituting a huge and complex dynamic balance system, which leads to the intestinal immune system having some regulatory mechanisms different from the systemic immune system and playing a crucial role in maintaining intestinal health (17, 18).

Caulis Spatholobi is the dried vine stem of the leguminous plant *Spatholobus suberectus* Dunn. Widely distributed in the Lingnan region and other places, it is a genuine medicinal herb. It has the effect of activating and hemostasis, regulating menstruation and relieving pain, relaxing tendons, and activating collaterals. Modern pharmacological studies show that suberect pathology not only has the function of promoting hematopoiesis but also has curative activaties of immuneoregulation, antitumor, antiviral, anti-inflammation, antioxidation, sedation, and hypnosis (19). The polysaccharide is one of the main bioactive components of Caulis pathologists. However, we found that there were few studies on the immunoactivity of Caulis Spatholobi polysaccharide (CSP) *in vivo*, and the correlation between immunosuppression and intestinal flora has not been fully studied in chickens. Therefore, this study was conducted to evaluate the regulatory effects of CSP on immunity, intestinal mucosal barrier function, and intestinal microbiota of cyclophosphamide-induced immunosuppression chickens and to provide experimental evidence for CSP as an immunoenhancer.

## Materials and Methods

### Materials and Chemicals

Caulis Spatholobi was purchased from the local market (Nanning, China). Cyclophosphamide was from Beyotime (Shanghai, China; 97.0–103.0%, reagent grade). Astragalus polysaccharide (APS) was purchased from Guilin Huayi Health Science and Technology Co., Ltd. (Guilin, China; purity ≥65%). Chicken IL-4, IL-10, IgG, IgM, TNF-β, Zo-1, and Occludin ELISA kits were purchased from Jiangsu Enzyme Immunity Industry Co., Ltd. (Yancheng, Jiangsu, China). The HE trial was conducted by Chengdu Lilai Biotechnology Co., Ltd. (Chengdu, China). TRIzol reagent, first-strand cDNA synthesis mix, and RealStar Green Fast Mixture were purchased from GenStar Biosolutions Co., Ltd. (Beijing, China). Molecular weight detection, monosaccharide composition detection, and infrared spectrum detection of CSP were performed by Qingdao Haida Marine Oligosaccharide Technology Co., Ltd. All other reagents in the experiment were purchased from China and were of analytical grade.

### Preparation of the Polysaccharide

The crushed Spatholobi was soaked in anhydrous ethanol (1:3, w/v). The ethanol was removed by extraction, and the residue was collected. Then, the CSP after degrees was dried in the bellows. After collecting the extract, vacuum evaporation, concentration, and filtration were performed. The obtained filtrate was treated with Sevag reagen (chloroform:*n*-butanol = 4:1. v/v) to remove proteins and with macroporous resin to remove pigments. Finally, 80% ethanol was added to the filtrate, and the filtrate was kept at 4°C for 24 h and centrifuged at 3,000 rpm for 20 min. The samples were precipitated with anhydrous ethanol and acetone and freeze-dried to obtain sample CSP initially.

### Determination of Physicochemical Properties of the Polysaccharide

The molecular weight was determined by high-performance gel permeation chromatography. The chromatographic column was a TSKgel GMPWXL, the standard curves of dexan with different molecular weights from China National Institute for The Control of Pharmaceutical and Biological Products were prepared, and the samples were detected. About 20 μL of 5 mg/ml sampleswas taken, according to the standard curve drawing method, for detection. The molecular weight was calculated by GPC software. The monosaccharide composition of samples was determined by the PMP precolumn derivatization method. Appropriate samples were weighed and completely hydrolyzed with TFA. The degradation solution was derivatized with PMP under alkaline conditions and then analyzed by HPLC after extraction with chloroform. The standard product does not need to be degraded but can be derivatized directly, and the derivatization method is the same as that for the sample. The peak area normalization method was used to calculate the monosaccharide composition of each sample. Appropriate amounts of samples were taken, and the KBr tablet method was used to conduct infrared spectrum analysis of the samples.

### Animal and Experimental Design

All animal testing protocols for this study were subjected to approval by the Experimental Animal Ethics Committee of Guangxi University (Nanning, China) (GXU-2021-162). A total of 288 1-day-old Sanhuang cocks (initial weight 32.77 ± 3.11 g) (Guangxi Fufeng Agriculture and Animal Husbandry Group Co., LTD, Nanning, China) were selected for this experiment. The chickens were raised in wired cages with good ventilation and lighting, and the relative humidity was maintained at 50–55%. From 1 to 14 days of age, the chicken house received 24 h light, and then the gradient decreased to 20 h light per day. At 1–7 days of age, the temperature of the chicken house was controlled at 32–34°C, and then the gradient was reduced to 26°C. During the whole experiment, the chickens were free to drink and eat. Chickens were randomly divided into six groups with six replicates per group and eight chickens per replicate: blank control group (N), cyclophosphamide model group (M), low-dose group (CLM), medium-dose group (CMM), high-dose group (CHM), and APS control group (ACM). The experiment lasted for 35 days. Cocks in groups N and M were fed a conventional diet, and cocks in CLM, CMM, and CHM groups were fed a conventional diet supplemented with 0.2, 0.4, and 0.6% CSP, respectively. The ACM group was given 0.4% APS on top of the standard diet. On days 8, 9, and 10, except for group N, the groups were intraperitoneally injected with 80 mg/kg BW cyclophosphamide, and group N was injected with an equal volume of normal saline. The weight of chickens was measured at 7, 14, 21, and 35 days of age (D7, D14, D21, and D35, respectively) after fasting for 2 h. Blood, spleen, thymus, bursa of Fabricius, jejunum, ileum, and cecum contents were collected for further analysis at D14, D21, and D35. The feed formula and nutrient level met the requirements of the National Research Council.

### Determination of Body Weight and Organ Indices

Chickens at the D14, D21, and D35 were weighed 2 h after fasting, and the spleen, thymus, and bursa of Fabricius were removed after euthanasia. Only after fat was removed and water was sucked up were the chickens were weighed accurately. The formula for the organ index is as follows: organ index (mg/g) = spleen or thymus weight (mg)/body weight (g).

### Determination of Cytokines, Immunoglobulins, and Tight Junction Proteins

The blood was collected in a sterile tube, and the blood coagulated naturally at room temperature for 10–20 min. The blood was centrifuged for 20 min (3,000 rpmat 4°C). Serum was collected, and the levels of IL-4, IL-10, IgG, IgM, TNF-β, Zo-1, and Occludin were detected by the ELISA kit. The detection method was performed as per the instructions provided by the ELISA kit manufacturer.

### Histopathological Staining

The jejunum and ileum of chickens were fixed with 10% neutral formaldehyde overnight and examined microscopically after dehydration, pruning, embedding, slicing, staining, and tablet sealing. The sections were observed under a digital trim camera microscope (BA210Digital, Motic China Group Co., Ltd.), and the villus length, crypt depth, and intestinal wall thickness of the jejunum and ileum tissue sections were measured and analyzed using image analysis software (Motic Images Advanced 3.2). A total of 10 groups of data for each index were measured. The average was calculated. The ratio of the villus length to crypt depth was calculated as an index to evaluate the degree of intestinal injury.

### RNA Extraction and RT-PCR Analysis

Depending on the manufacturer's instructions (GenStar Biosolutions Co., Ltd, Beijing, China), 50–100 mg of frozen jejunum samples was ground in liquid nitrogen, and RNA was extracted with TRIGene total RNA extraction reagent. The concentration of RNA in the sample was determined by a nucleic acid protein analyzer, and the D260/D280 of the detected RNA met the reverse transcription conditions in the range of 1.8–2.0. StarScript II First-Strand cDNA Synthesis Mix (GenStar Biosolutions Co., Ltd, Beijing, China) with gDNA remover was used to synthesize cDNA. The PCR reaction was performed on a LightCycler 96 System (Roche) using the RealStar Green Fast Mixture. Using β-actin as internal reference, the expression levels of *TLR4, MyD88, NF-*κ*B, Claudin-1*, and *Zo-1* genes were detected by RT-PCR, and the relative expression levels of these genes were calculated by the 2^−ΔΔCT^ method. The sequences of the primers used in the present study are listed in Supplementary Table S1. The primer sequences used in this study (Sangon Bioengineering Co., Ltd, Shanghai, China) are given in the Supplementary Table S1.

### Intestinal Flora DNA Extraction and High-Throughput Sequencing of 16S RRNA Gene

DNA of cecal contents was extracted using the DNA kit (Omega Bio-Tek Inc., Norcross, GA, USA). The extracted DNA was identified by 1% agarose gel electrophoresis and spectrophotometry (260/280 nm optical density ratio). The V3-V4 extender primers of 16S rDNA were 338F (5′-ACTCCTACGGGAGgCAGCAGcag-3′) and 806R (5′-GGACTACNNGGG TATctaat-3′). The V3-V4 region of bacterial 16S rDNA was selected and sequenced by an Illumina (Illumina, Inc., San Diego, CA, USA) Miseq PE300 high-throughput sequencing platform. The off-machine data were handled by QIIME (V1.8.0) software. The sequence information of each processing group is clustered into operational taxonomic units (OUTs) for species classification according to barcodes. Mothur software (version 1.31.2) was used for α diversity analysis. Based on the weighted UniFrac distance, the pheatmap package of R (V3.1.1) software was used for cluster analysis. The UniFrac algorithm was utilized to compare species community differences between samples using the information of systematic evolution, and the beta diversity analysis was performed. Linear discriminant analysis (LDA) and linear discriminant analysis effect size (LEFSE) were used to analyze the microbial community dominance between groups. The original sequence was uploaded to NCBI's Sequence Read Archive database.

### Statistical Analysis

All data were expressed as mean ± standard deviation, and at least three independent experiments were conducted. SPSS version 22 (IBM, Armonk, New York, USA) and Graph Pad Prism version 8 (Graph Pad Software Inc., San Diego, CA, USA) were used to analyze and process data. Differences among groups were analyzed by one-way analysis of variance and Duncan's multirange test, and *p* < 0.05 was considered statistically significant.

## Results

### Physicochemical Properties of CSP

**Figure 1:**
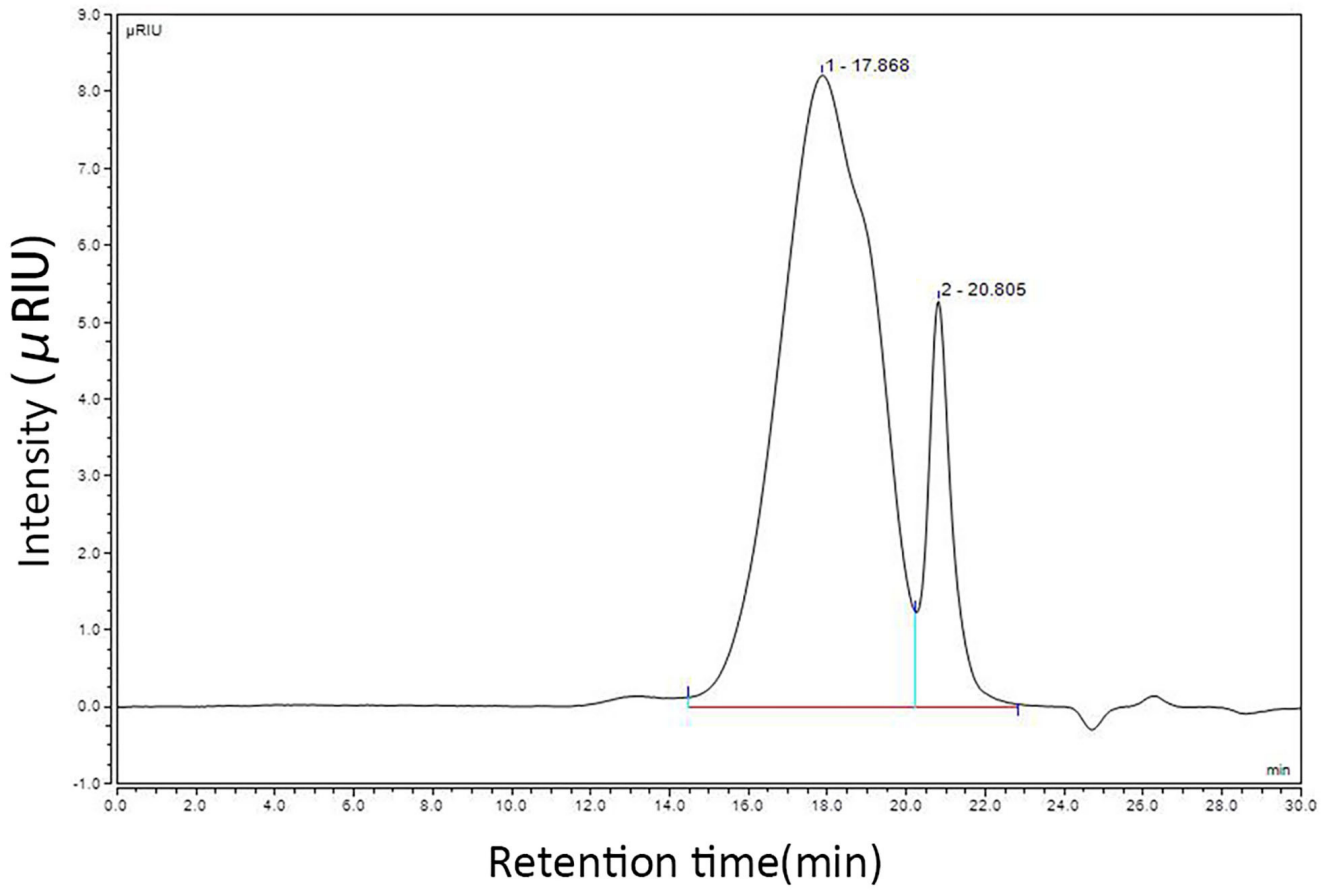
Molecular weight detection results of CSP.

**Figure 2:**
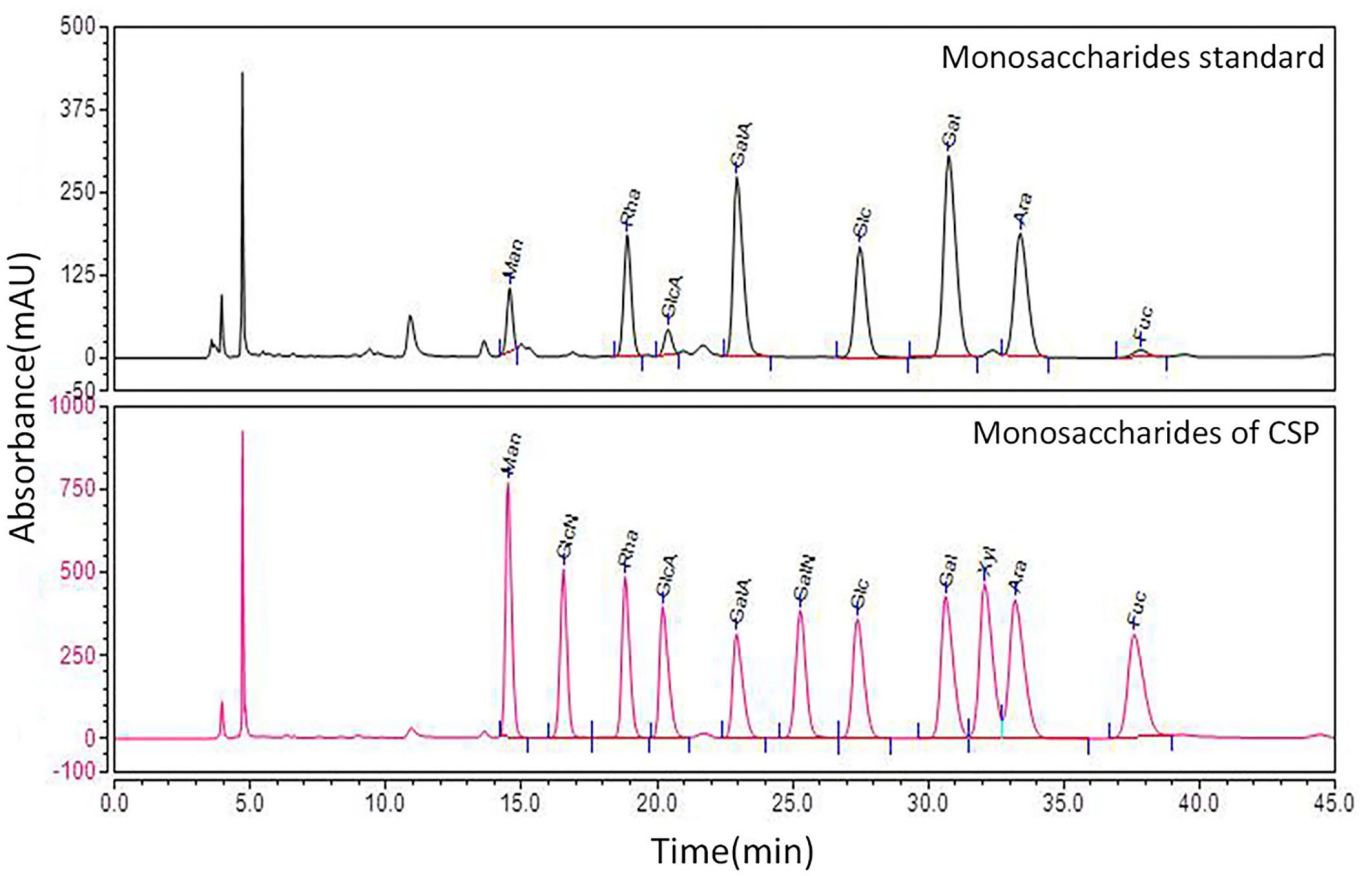
Monosaccharide composition analysis HPLC chromatogram.

Using the CSP preparation method mentioned in this study, the extraction rate of CSP was calculated to be 3% and the sugar content of CSP was 80% (phenol sulfuric acid method). To prove that the CSP used in this study has the basic characteristics of polysaccharides, it was isolated and purified using DEAE cellulose 52, and the physicochemical properties of purified CSP were detected. However, this study paid more attention to the practical clinical application value, so crude polysaccharide was used in the follow-up test. Calculated by D0 glucan, the number of theoretical plates is higher than 5,000, indicating a good column effect. The standard curve equation is *Y* = −0.5198*x* + 13.549, *R*^2^ = 0.9935, indicating a good linear relationship. According to the HPLC instrument integral, the chromatographic peak is shown in Figure 1. The results show that the peak time of CSP is 17.868 min, and the mobile phase is 20.805 min. The average molecular weight (*M*_w_) and distribution width (PD) of CSP were 38.5 kDa and 4.81, respectively. The large PD value indicates that the sample is not uniform. Monosaccharide composition analysis of CSP samples was determined by the PMP precolumn derivatization method. HPLC chromatograms of CSP samples are shown in Figure 2.

**Figure 3:**
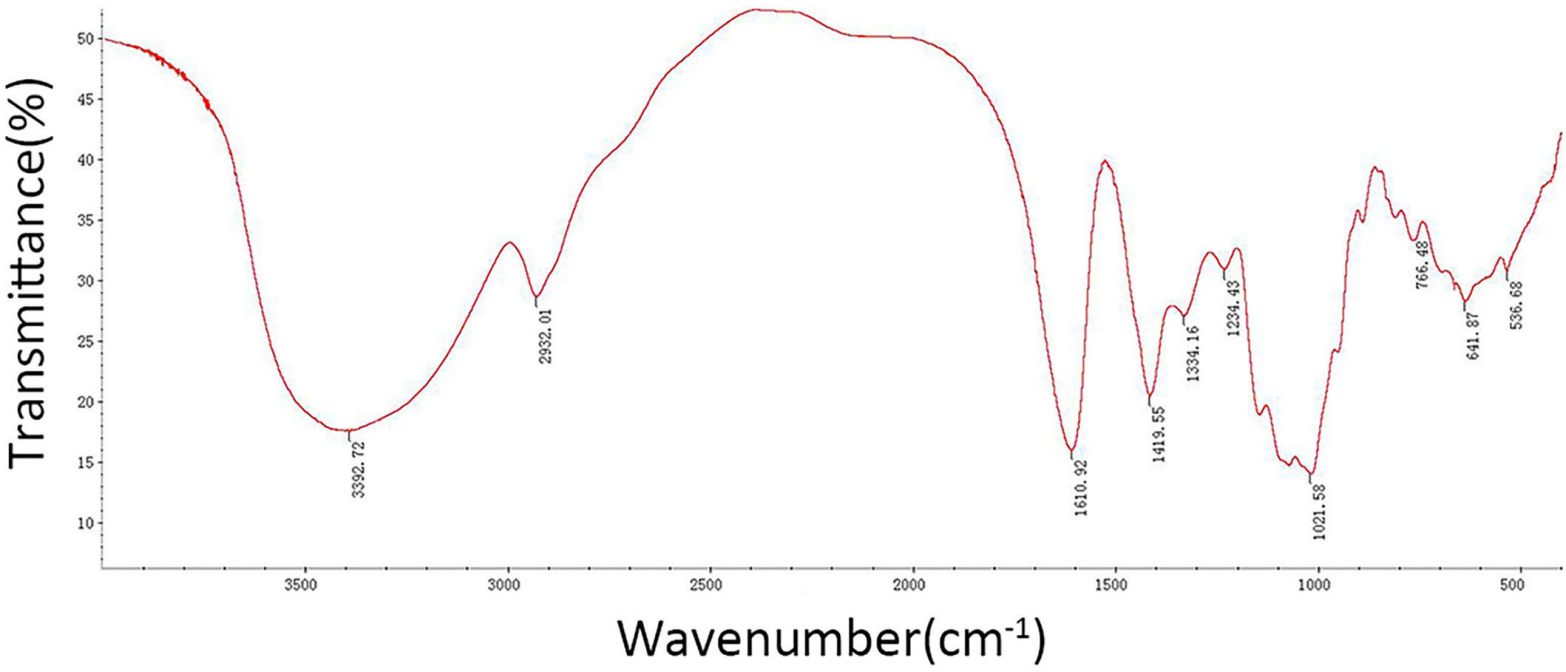
FT-IR spectra of CSP.

The results showed that the CSP samples were mainly composed of Man (7.7%), Rha (14.6%), GlcA (3.1%), GalA (21.6%), Glc (13.3%), Gal (24.2%), Ara (14.8%), and Fuc (0.8%). The CSP sample was scanned by infrared spectroscopy, and the determined infrared spectrum is given in Figure 3. At 34,000 cm^−1^, the characteristic absorption peak of sugar ring C– H was observed. At 1,600 cm^−1^, the characteristic absorption peak of the carbonyl, aldehyde, or carboxyl groups was observed. At 1,200–1,400 cm^−1^, the characteristic absorption peak of sugar ring C–H was observed. The palm-like peak at 1,000–1,200 cm^−1^ was the absorption peak of the pyranose ring. The infrared spectra of CSP samples accord with the basic characteristics of the infrared spectra of polysaccharides.

### Effects of CSP on Body Weight and Immune Organ Index of Immunosuppressed Chickens

We evaluated the success of this experimental model by recording the changes in the body weight (BW) and immune organ index of chickens before and after CY injection and analyzed the effects of CSP on the BW and immune organ index of chickens of each group (Table 1). No chickens died during the trial. At D14, D21, and D35, the BW of chickens in group M was significantly lower than that in group N (*p* < 0.05). At D14, the thymus index and the bursa of Fabricius index in group M were markedly lower than those in group N (*p* < 0.01). These results indicated that the establishment of the immunosuppressed chicken model is successful. At D14 and D21, the BW of chickens in the CMM and ACM groups was much higher than that in the M group (*p* < 0.01). At D35, the BW of CLM and CMM groups was significantly higher than that in the M group (*p* < 0.01). At D14, the thymus index in the ACM group and the bursa of Fabricius index in the CLM group were significantly higher than those in the M group (*p* < 0.01). At D21, the bursa of Fabricius index in the CHM group and ACM group was significantly higher than that in the M group (*p* < 0.05). At D35, the spleen index in the CMM group was notably higher than that in the M group (*p* < 0.01). These results indicated that CSP can effectively improve the weight loss induced by cyclophosphamide, and there was no significant difference between the immune organ index of each group and that of the M group, but the index showed an increasing trend.

**Table 1:** Effects of CSP on body weight and immune organ index of immunosuppressed chickens.

**Traits**	**Age (d)**	**Treatment**					
		**N**	**M**	**CLM**	**CMM**	**CHM**	**ACM**
BW (g)	7	51.38 ± 5.77	52.96 ± 3.80	54.02 ± 9.23	50.96 ± 8.06	51.31 ± 6.17	52.70 ± 8.43
	14	94.46 ± 12.09	62.34 ± 3.16^**^	65.23 ± 6.95^**^	85.00 ± 6.06^*^^##^	65.29 ± 6.34^**^^*^	85.70 ± 7.84^##^
	21	128.06 ± 12.39	112.61 ± 7.06^*^	126.97 ± 8.96^#^	145.51 ± 12.81^**^^##^	124.17 ± 12.30	140.88 ± 7.23^*^^##^
	35	326.80 ± 12.91	259.47 ± 14.89^**^	303.85 ± 33.77^##^	309.80 ± 41.47^##^	233.08 ± 16.89^**^	256.43 ± 30.97^**^
Spleen index (mg/g)	14	1.92 ± 0.57	2.60 ± 0.53	1.77 ± 0.50	3.66 ± 1.57^**^	2.09 ± 0.42	2.33 ± 0.19
	21	3.11 ± 0.27	2.13 ± 1.17	1.86 ± 0.25	1.97 ± 0.42	3.03 ± 1.53	1.88 ± 0.44
	35	2.10 ± 0.41	1.44 ± 0.27	1.99 ± 0.32	2.81 ± 0.73^^*^^##^^	1.71 ± 0.30	2.12 ± 0.49
Thymus index (mg/g)	14	2.68 ± 0.68	0.61 ± 0.28^**^	1.06 ± 0.44^**^	1.23 ± 0.59^**^	0.84 ± 0.50^**^	1.98 ± 1.01^##^
	21	2.19 ± 0.27	3.41 ± 0.87	3.85 ± 0.88^*^	2.81 ± 0.54	2.05 ± 0.48	2.28 ± 1.65
	35	5.37 ± 2.35	4.57 ± 0.78	4.39 ± 1.54	5.64 ± 0.90	5.57 ± 2.23	4.36 ± 1.48
Bursa of Fabricius (mg/g)	14	3.40 ± 0.69	0.89 ± 0.19^**^	2.56 ± 1.97^##^	0.93 ± 0.26^**^	0.85 ± 0.10^**^	1.00 ± 0.45^**^
	21	2.46 ± 1.02	2.48 ± 0.29	2.96 ± 0.90	1.59 ± 0.21	1.03 ± 0.68^*^^#^	1.06 ± 0.29^*^^#^
	35	3.52 ± 0.72	0.94 ± 0.28^**^	1.18 ± 0.23^**^	1.21 ± 0.46^**^	0.89 ± 0.18^**^	0.95 ± 0.30^**^

*Data are expressed as mean ± SD*.

* *p < 0.05*,

** *p < 0.01 ompared with N roup*;

# *p < 0.05*,

## *p < 0.01 compared with M group*.

### Effects of CSP on the Immune Function of Immunosuppressed Chickens

**Figure 4:**
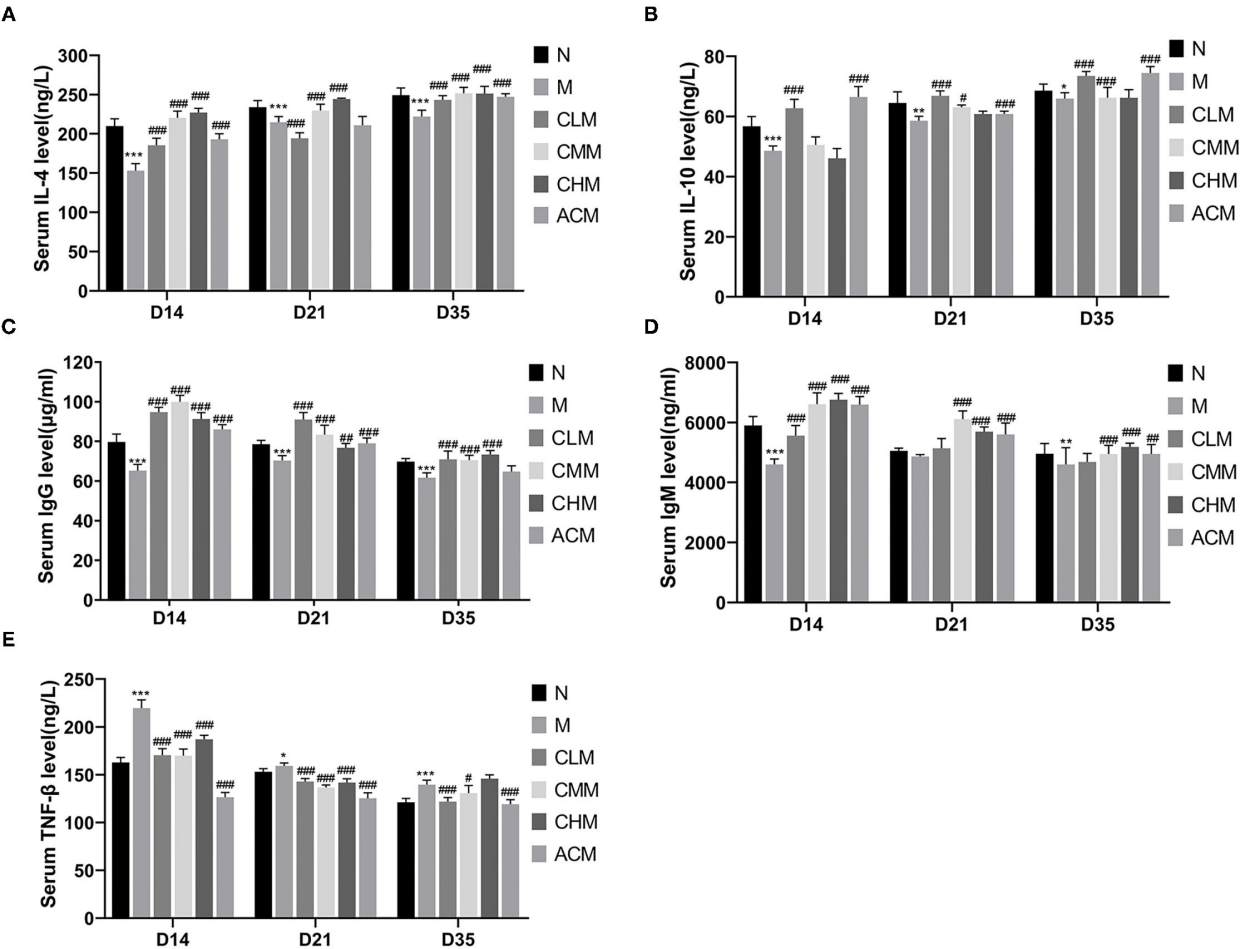
Effects of CSP on the levels of IL-4 **(A)**, IL-10 **(B)**, IgM **(C)**, IgG **(D)** and TNF-β **(E)** in serum of chicken. Data are expressed as mean ± SD.*p < 0.05, **p < 0.01, and ***p < 0.001 compared with N group; ^#^*p* < 0.05, ^##^*p* < 0.01, and ^###^*p* < 0.001 compared with M group.

We evaluated the effect of CSP on the immune function of cyclophosphamam-induced immunosuppression chickens by measuring cytokines, immunoglobulin and tumor necrosis factor (Figure 4). The serum LEVELS of IL-4, IL-10 and IgG in the M group were significantly lower than those in the N group at D14 (*P* < 0.05), D21 (*P* < 0.01) and D35 (*P* < 0.01) days of age. And the serum IgM level in M group was also significantly lower than that in N group at D14 (*P* < 0.01) and D35 (*P* < 0.01) days of age. These results suggest that CSP can inhibit the immune activity of the body. Meanwhile, compared with group M, the results of each administration group were as follows: the serum LEVELS of IL-10 and IgG in the CLM group were significantly increased at D14, D21 and D35 days of age (*P* < 0.01), while the serum levels of IL-4, IgM and IgG in CHM group were significantly increased at 14, 21 and 35 days of age (*P* < 0.01). The serum LEVELS of IL-10 and IgM in the ACM group also increased significantly at D14, D21 and D35 days of age (*P* < 0.01). However, the content of TNF-β in the M group was significantly higher than that in the N group at 14, 21 and 35 days of age (*P* < 0.05). Compared with the M group, the content of TNF-β in the CLM group and CMM group was significantly decreased (*P* < 0.05). These results fully suggest that CLM, CMM, CHM and ACM groups can restore CY induced immunosuppression and improve the immune function of chickens by promoting the production of cytokines IL-4, IL-10, immunoglobulin IgM and IgG and inhibiting the increase of TNF-β content induced by CY.

### Effects of CSP on the Intestinal Mucosal Barrier in Immunosuppressed Chickens

**Figure 5:**
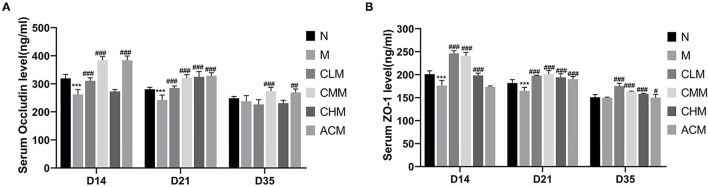
Effects of CSP on serum and Occludin **(A)** and Zo-1 **(B)** levels. Data are expressed as mean ± SD. ***p < 0.001 compared with N group; ^#^*p* < 0.05, ^##^*p* < 0.01, and ^###^*p* < 0.001 compared with M group.

**Figure 6:**
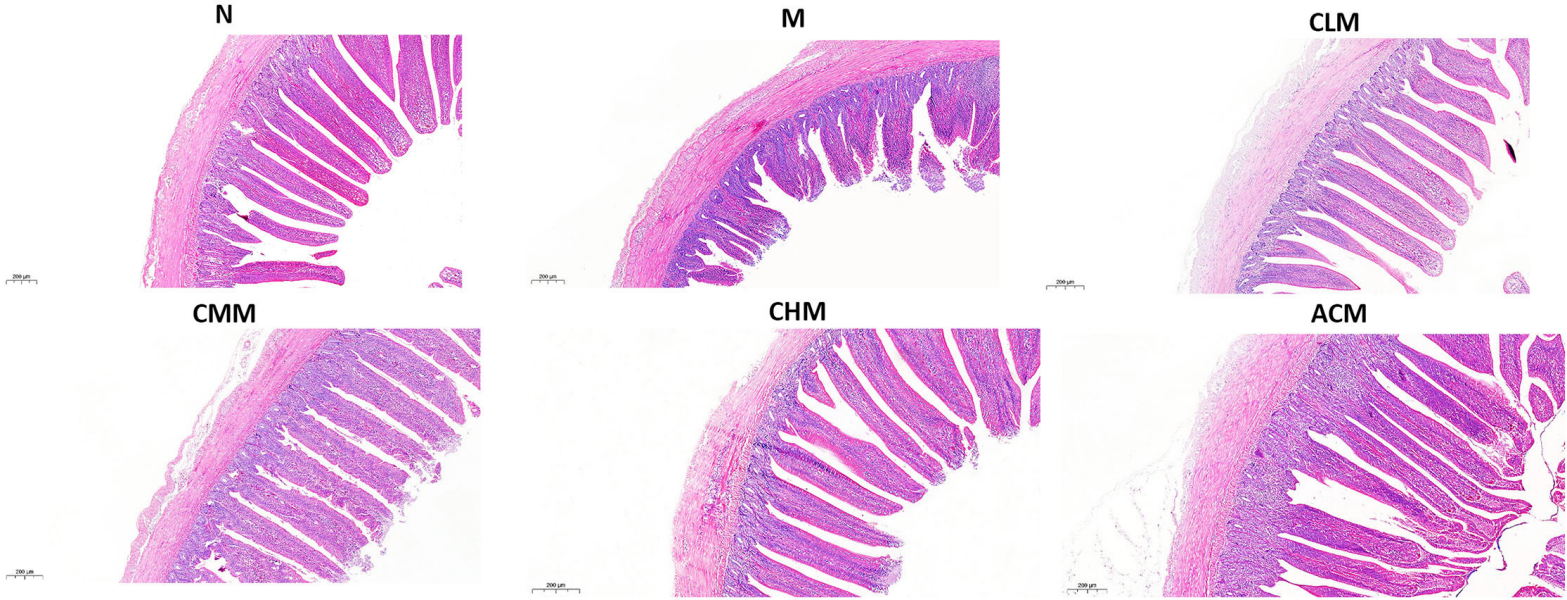
Effects of CSP on intestinal murphology of chicken ileum H&E (100x, Scale bar = 200 mm).

**Figure 7:**
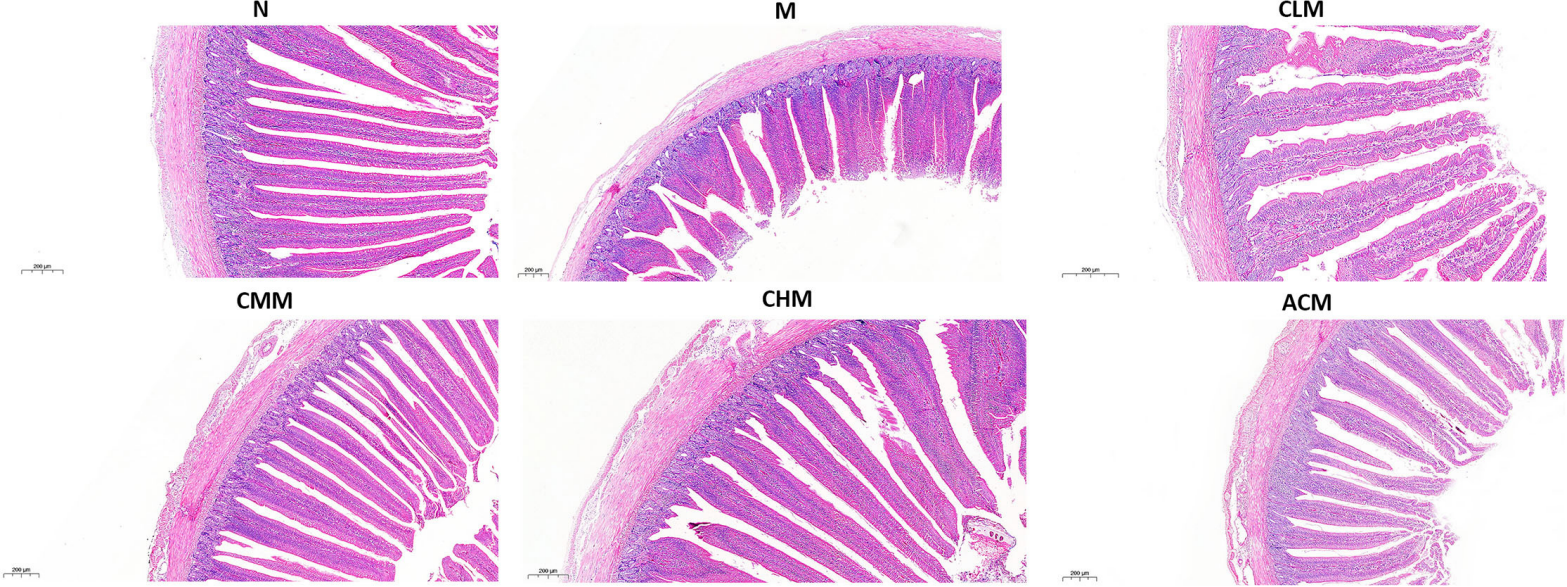
Effects of CSP on intestinal murphology of chicken jejunum H&E (100x, Scale bar = 200 mm).

Tight junction is the main connection mode between intestinal epithelial cells, which play a major role in the mechanical integrity and normal function of the intestinal mucosal barrier. First, the concentrations of tight junction proteins Zo-1 and Occludin in the M group were significantly lower than those in the N group at D14 and D21 (*p* < 0.01), and the concentrations of Zo-1 in the CLM, CMM, and CHM groups were markedly higher than those in the M group at D14, D21, and D35 (*p* < 0.01). Similarly, the occludin contents in CLM and CMM groups were substantially higher than those in the M group at D14 and D21 (*p* < 0.01; Figures 5A,B). In conclusion, CSP protects the intestinal mucosal barrier by increasing the expression levels of tight junction proteins Zo-1 and Occludin. HE staining results are shown in the figure: the ileum and jejunum of chickens in group N had complete morphology, orderly arrangement of intestinal villi, complete structure, fine structure and shallow crypt. The intestinal structure of chickens in group M was damaged, the intestinal villi were broken, atrophied and coarsed, while the morphology of ileum and jejunum after CSP treatment were similar to those in group N, and the intestinal injury symptoms were recovered (Figures 6, 7).

**Figure 8:**
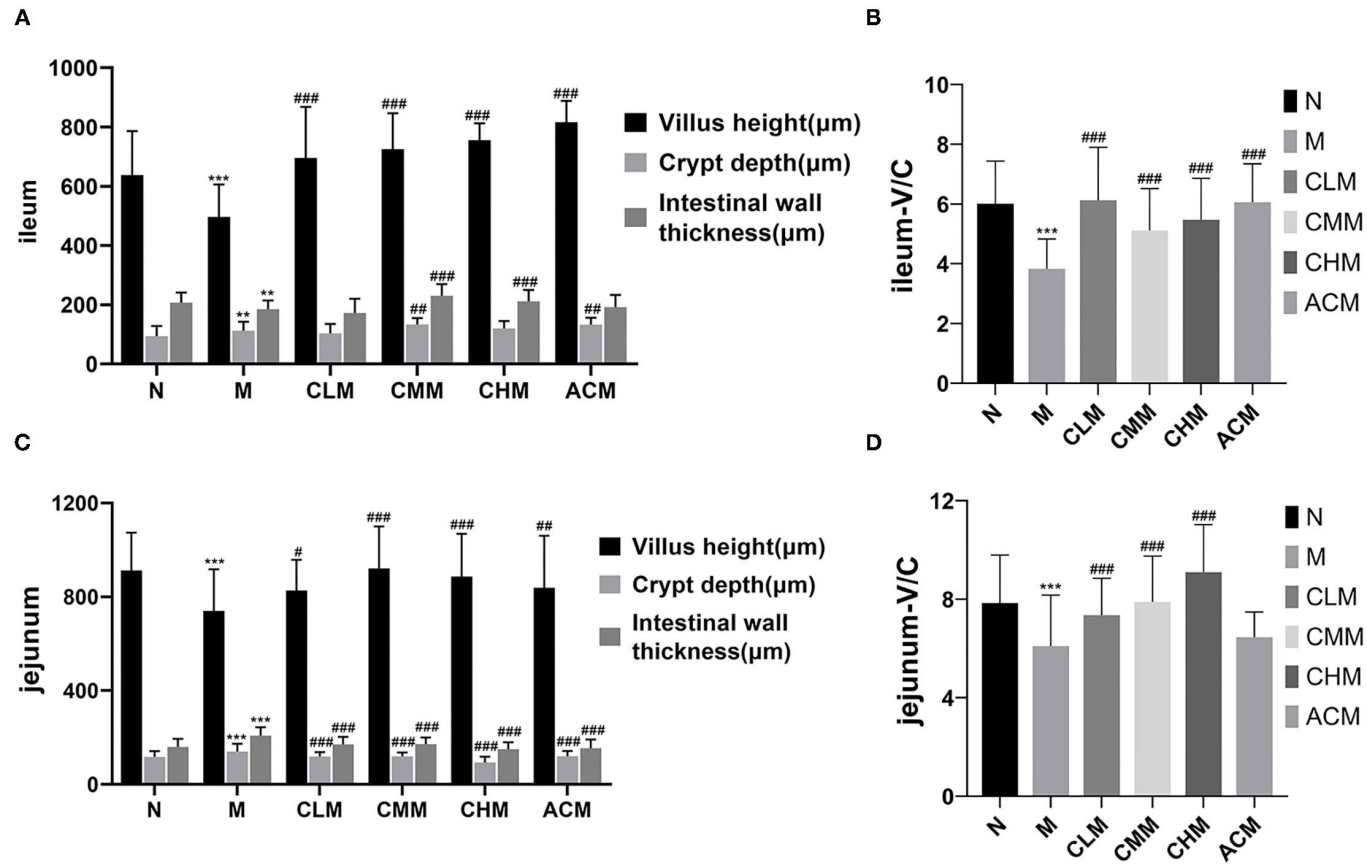
Effects of CSP on villus height, crypt depth and wall thickness of ileum **(A)**, V/C ratio of ileum **(B)**, villus height, crypt depth and wall thickness of jejunum **(C)** and V/C ratio of jejunum **(D)** in chicken intestine. Data are expressed as mean ± SD. **p < 0.01, and ***p < 0.001 compared with N group; ^#^*p* < 0.05, ^##^*p* < 0.01, and ^###^*p* < 0.001 compared with M group.

According to the analysis report of jejunum and ileum measurements, the following results are obtained. The villus height of the ileum and jejunum in group M was significantly lower than that in group N, the crypt depth was significantly higher than that in group N, and the intestinal wall thickness was significantly higher than that in group N (*p* < 0.01). The intestinal villus height of the ileum and jejunum in CLM, CMM, CHM, and ACM groups was significantly higher than that in the M group, while the intestinal crypt depth and intestinal wall thickness of the jejunum in CLM, CMM, CHM, and ACM groups were significantly lower than those in the M group (*p* < 0.05, *p* < 0.01). In addition, the V/C ratio in CLM, CMM, and CHM groups was significantly higher than that in the M group (*p* < 0.01). These results suggested that CSP can increase the intestinal villus height and decrease crypt depth, thereby increasing the V/C ratio and protecting the intestinal mucosal function (Figure 8).

### Effects of CSP on the Expression of Relating Genes in the Jejunum of Immunosuppressed Chickens

**Figure 9:**
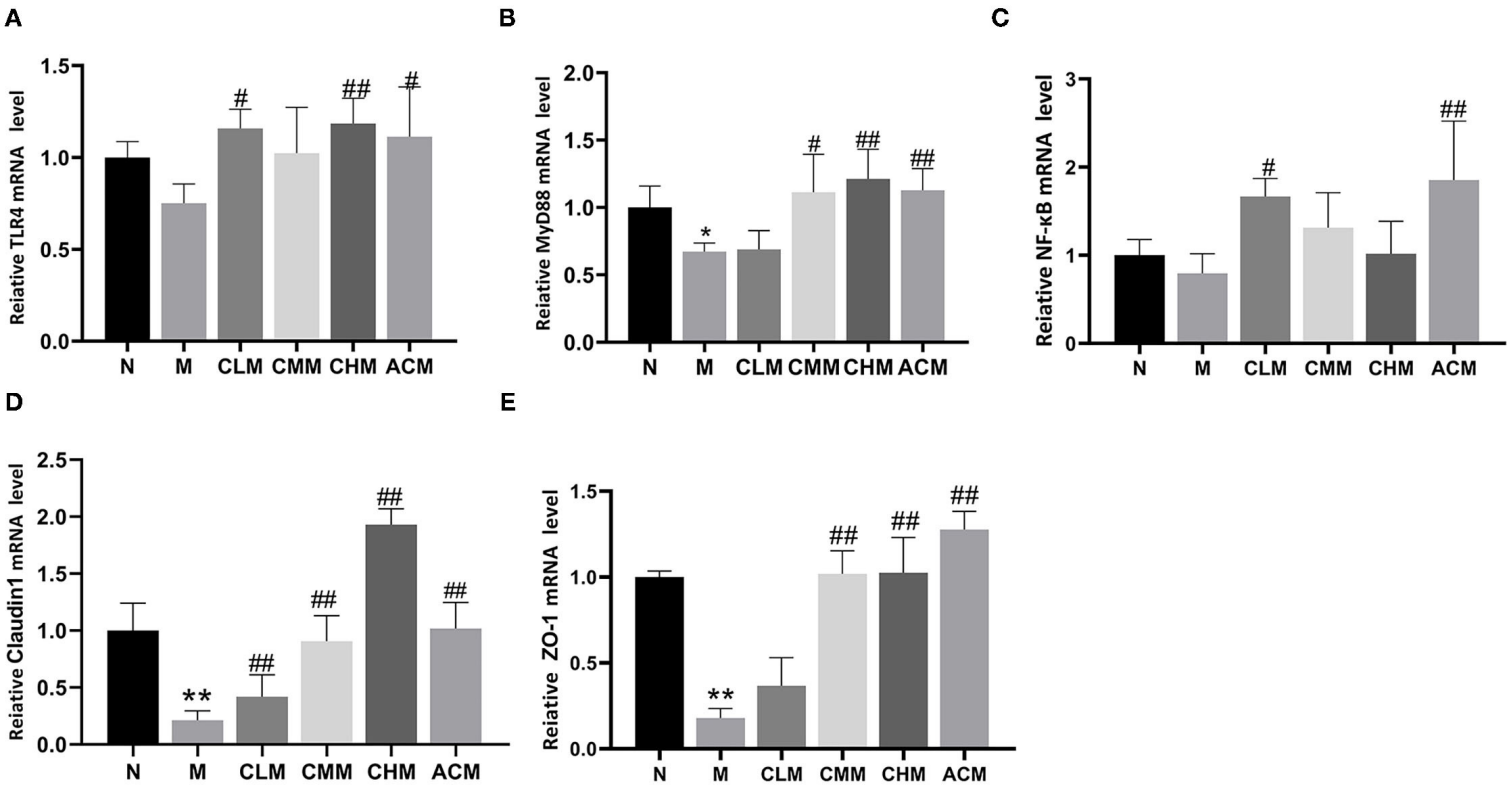
Effects of CSP on relative mRNA expression in jejunum. **(A)** The mRNA expression of TLR4. **(B)** The mRNA expression of MyD88. **(C)** The mRNA expression of NF-κB. **(D)** The mRNA expression of Claudin1. **(E)** The mRNA expression of Zo-1. Data are expressed as mean ± SD.*p < 0.05 and **p < 0.01 compared with N group; ^#^*p* < 0.05 and ^##^*p* < 0.01 compared with M group.

To further explore the effect mechanism of CSP on the intestinal mucosal barrier, the relative expression levels of *TLR4, MyD88, NF-*κ*B, Claudin1*, and *Zo-1* genes in the jejunum were further studied in this study (Figure 9). First, in terms of the mRNA expression level, the expression level of the MyD88 gene in the M group was significantly downregulated (*p* < 0.05), followed by the expression level of TLR4 and NF-κB in the M group (no statistical difference). Interestingly, the expression levels of TLR4, MyD88, and NF-κB were significantly upregulated in CLM and ACM groups compared with those in the M group (*p* < 0.05, *p* < 0.01). Meanwhile, the expression levels of MyD88 in the CMM group and TLR4 and MyD88 in the CHM group were also significantly upregulated (*p* < 0.05). As shown Figures 9D,E, CY resulted in significant downregulation of tight junction protein *Claudin1 and Zo-1* genes in the jejunum (*p* < 0.01). However, the CSP reversed this phenomenon: CLM, CMM, CHM, and ACM groups showed significantly upregulated Claudin1 gene expression, and the other groups except CLM also showed significantly upregulated Zo-1 gene expression (*p* < 0.01). In conclusion, the CSP can upregulate the expression levels of *TLR4, MyD88, NF-*κ*B, Claudin1, and Zo-1* genes in the jejunum and improve colonic mucosal injury.

### Effects of CSP on Intestinal Microflora of Immunosuppressed Chickens

**Figure 10:**
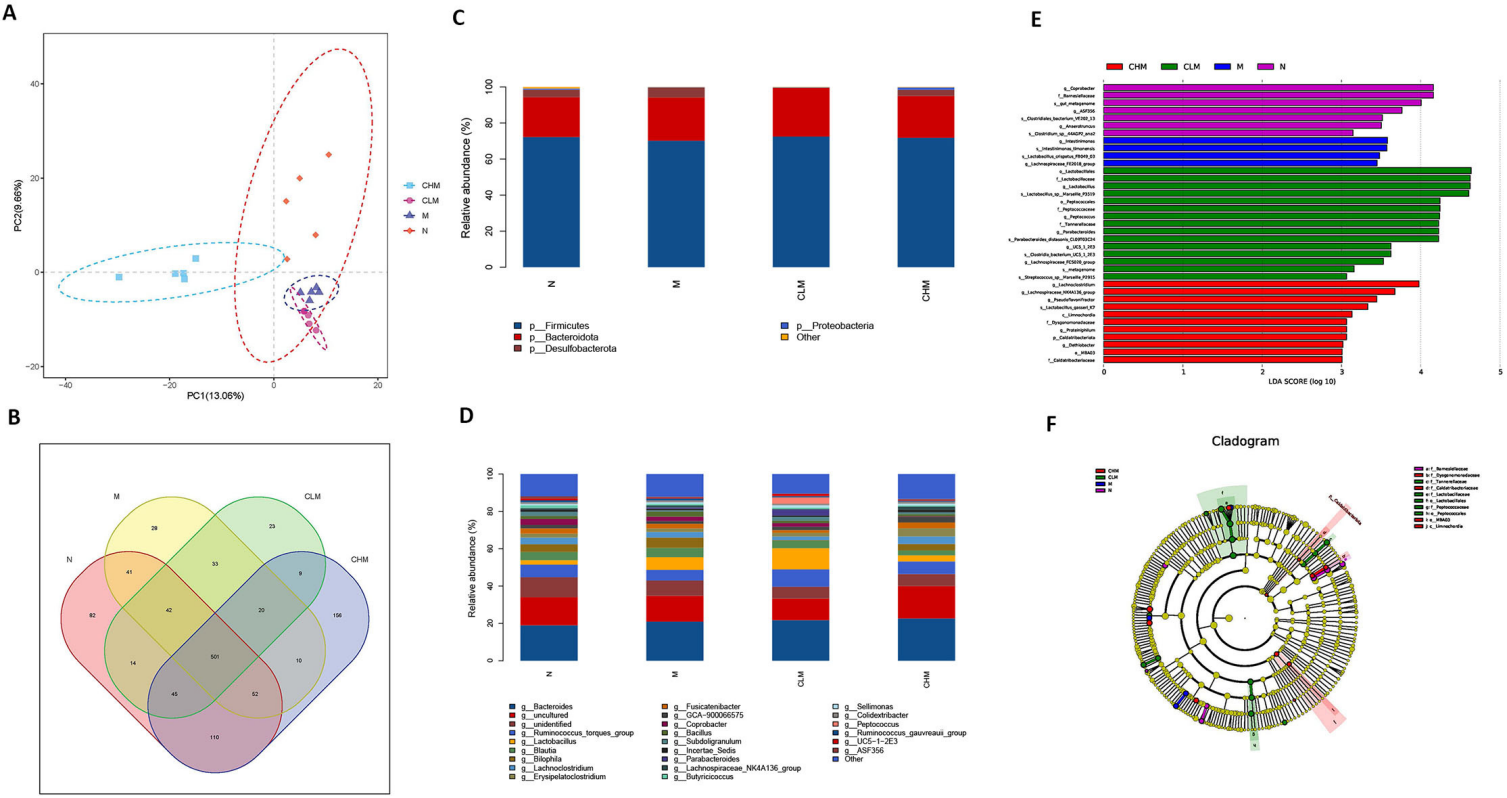
Effects of CSP on intestinal microflora of immunosuppressed chickens. **(A)** Partial least squares discriminant analysis (PLS-DA). **(B)** Venn diagram. **(C,D)** The abscissa is the sample name, and the ordinate is the relative abundance of species in the sample. The graph shows information about species with relative abundance of more than 1%. **(C)** Microbe distribution at phylum level in different groups. **(D)** The distribution of microorganisms at the generic level in different groups. **(E)** Histogram of LDA ditribution based on LEfSe analysis of classification information. **(F)** Evolutionary branching diagram of LEfSe analysis based on classification information.

Cecal feces of chickens at D35 were selected for high-throughput gene sequencing analysis of 16S rDNA. First, according to the results of the rarefaction curve, Shannon–Wiener, rank-productivity curve, and species accumulation curve, the sequencing depth and the number of samples were reasonable and reliable (Supplementary Figure S1). The abundance and diversity of microbial communities were determined by alpha diversity analysis. A series of statistical analysis indices were used to summarize the results, as follows: In the OTU, Simpson, Chao1, and Shannon indices, compared with the N group, cyclophosphamide reduced the species diversity of intestinal fecal contents (*p* < 0.05, *p* < 0.01). Surprisingly, CSP increased OTU, Simpson, Chao1, and Shannon indices and increased the species diversity of microbial community (Table 2). In addition, the similarities or differences in the composition of the sample communities were analyzed through beta diversity analysis. The results of PLS-DA analysis (Figure 10A), namely, partial least square discriminant analysis, showed that the microbial community structure of CHM and N groups was significantly different from that of the M group, while that of the CLM group was similar to that of the M group. Similar observations can be obtained in NMDS analysis and system cluster analysis. To more intuitively observe the OTUs of samples and the overlap between the samples or groups, a Venn diagram was drawn (Figure 10B). As shown in the figure, there were 501 OTUS in the four groups. The unique OTUs of N, M, CLM, and CHM groups were 82, 28, 23, and 156, respectively, indicating that species abundance was higher in the CHM group.

**Table 2:** α-diversity indices of gut microbiota in each group.

**Groups**	**OTUs**	**Simpson**	**Chao1**	**AACE**	**Shannon**
N	534.50 ± 36.89	0.96 ± 0.01	737.76 ± 57.86	692.74 ± 128.87	6.07 ± 0.31
M	423.50 ± 70.79^**^	0.91 ± 0.01^**^	560.18 ± 71.21^*^	619.80 ± 109.30	5.43 ± 0.39^*^
CLM	438.80 ± 38.35^*^	0.92 ± 0.02^*^	594.20 ± 57.61^*^	581.87 ± 54.73	5.46 ± 0.30^*^
CHM	574.80 ± 22.88	0.92 ± 0.03^*^	805.39 ± 39.68^##^	830.45 ± 44.15	5.68 ± 0.38

*Data are expressed as mean ± SD*.

* *p < 0.05*,

** *p < 0.01 ompared with N roup*;

## *p < 0.01 compared with M group*.

To further study the effect of CSP on microbial community composition, the classification levels of each group were compared in detail. At the phylum level (Figure 10C), Firmicutes and Bacteroidetes are the two most dominant phyla in the microflora. The abundance of Firmicutes decreased in the M group, but there was no significant difference at the phylum level among the four groups. To further verify the differences in intestinal flora in each group, further analysis was performed at the genus level (Figure 10D): the N and CHM groups were mainly composed of Bacteroides, Ruminococcus_torques_group, and Bilophila. Group M was primarily composed of Bacteroides, Lactobacillus, and Bilophila. Compared with the N group, the relative abundance of Ruminococcus_torques_group, Barnesiella, and Anaerotruncus in the M group decreased. The relative abundance of Bilophila, Barnesiella, and Anaerotruncus in the CLM group decreased compared with that in the N group. There were differences in species abundance among N, M, CLM, and CHM groups, but the differences were not significant. LDA effect size (LefSe) analysis can be used to compare multiple groups. LefSe analysis can be used to determine the microflora in different groups of the intestinal tract. Finally, LDA (>3 was used to reduce the dimension of data to evaluate the influence of significantly different species. According to Figures 10E,F, it can be seen that the LDA scores of Coprobacter, Barnesiellaceae, Gut_Metagenome, and ASF356 are relatively high, indicating that there are abundant OTUS in group N. However, the SCORES of GROUP M were higher: Intestinimonas_timonensis, a Lactobacillus_crispatus_FB049_03, and a Lachnospiraceae_FE2018_group. These species may be more susceptible to cyclophosphamide stimulation. From the composition of the CLM group, Lactobacillales, Lactobacillaceae, Lactobacillus, Lactobacillus_sp__Marseille_P3519, and Peptococcales recovered to a high level. In the CHM group, Lachnoclostridium, Lachnospiraceae_NK4A136_group, Pseudoflavonifractor, and Lactobacillus_gasseri_K7 also recovered to high levels, suggesting that CSP greatly increased the increment of the microbial Lactobacillus *in vivo* and thus alleviated the CY-induced immunosuppression.

## Discussion

CY is a chemotherapy drug and is often used as an immunosuppressant, which inhibits humoral and cellular immunity, killing tumor cells and causing serious side effects. CY can lead to a loss of immune function and impaired intestinal function, increasing the likelihood of secondary infection (2, 14, 20). Therefore, cyclophosphamide was used in this study to establish a chicken immunosuppressive model and to evaluate the immunoregulatory effect of CSP on immunosuppressive chickens. The results showed that CY resulted in significantly lower BW, immune organ index, cytokines, immunoglobulin, tight junction protein, and intestinal mucosal barrier function than the normal group, indicating that cyclophosphamide had a significant inhibitory effect on the immune function and intestinal mucosal barrier in chickens. Therefore, the immunosuppression model of chicken established in this study was successful.

Immunoactivity is regarded as one of the most important defense strategies for preventing and fighting infection, inflammatory diseases, and cancer (21). The thymus, spleen, and bursa of Fabricius are important immune organs of chickens. The organ index determines the degree of lymphocyte proliferation, which can reflect the strength of immune function to a certain extent (22, 23). The thymus and bursa of Fabricius are important central immune organs, and their main function is to produce T lymphocytes and secrete thymosin, which is mainly involved in cellular immunity. The spleen is a peripheral immune organ, which is rich in lymphocytes and macrophages and is mainly engaged in humoral rabbit disease. The level of the immune organ index can take into account the degree of lymphocyte proliferation (20). Studies have shown that some natural plant polysaccharides can improve the immune organ index of chickens and inhibit the atrophy of immune organs (8, 24, 25). We observed that CY significantly inhibited the BW and immune organ index, while CSP alleviated this situation to a certain extent, suggesting that CSP can enhance the immune function of chickens by stimulating the development of immune organs. However, we also observed that the inhibition of cyclophosphamide to chickens recovered to some extent with the prolongation of the time of cyclophosphamide *in vivo* after modeling. This suggested that the inhibitory effect of CY on the BW and immune organ index was not persistent (26, 27).

A large number of studies have shown that polysaccharides can act on immune cells, participate in the regulation of innate and adaptive immune responses, and play an important role in the occurrence and development of infectious diseases, tumors, and immune-related diseases (28, 29). *Achyranthes bidentata* polysaccharides can promote the proliferation of spleen lymphocytes in a dose-dependent manner and play an immunomodulatory role through IL-2 and TNF-α (30). Taishan Pinus massoniana pollen polysaccharide and propolis can improve the immune organ index and expression of immune-related genes IL-2, IL-6, and IFN-γ to protect immune organs (13). IL-4, a cytokine secreted by Th2 cells, plays a major role in the regulation of humoral immunity and adaptive immunity (31). IL-10 has a bidirectional immunomodulatory effect, which can be suppressed by antigen presenting cells and negatively regulated by T cells (32). In our study, CY significantly inhibited the production of cytokines IL-4 and IL-10 and immunoglobulins IgM and IgG. Surprisingly, serum levels of cytokines IL-4 and IL-10 and immunoglobulins IgM and IgG were significantly increased in all low, medium, and high doses of CSP and 14, 21, and 35 days of CSP feeding. CY significantly increased the serum TNF-β content, while CSP decreased the serum TNF-β content to the normal level. These results indicate that CSP regulates the immunosuppressive state by increasing the secretion levels of cytokines (IL-4, IL-10) and immunoglobulins (IgM, IgG) and decreasing the secretion level of TNF-β. The results showed that CSP restored the immune capacity of chickens and improved the immune response level of the host. Intestinal mucosal injury is one of the main toxic side effects of CY (14). The intestinal tract is an important immune organ of the body and plays an important role in the microflora and immune system, so there is a close relationship between intestinal immunity and systemic immune response (33, 34). The villus height and crypt depth of the small intestine are important indices to the digestive and absorption capacity of the small intestine. The ratio of the villus height to crypt depth can comprehensively reflect the functional status of the small intestine, and the decreased ratio indicates that the mucosa is damaged and the digestion and absorption ability is decreased (35). Therefore, we detected the intestinal villus height, crypt depth, and intestinal wall thickness, calculated the V/C ratio, and observed intestinal mucosal injury by intestinal HE staining, thus evaluating the effect of CSP on the intestinal mucosal barrier. The first thing to note is that the intestinal integrity of chickens changes with age (36). The study also showed that the species richness of cecum contents of chickens was the highest and gradually remained stable after D21. Therefore, ileum and jejunum tissues and cecum contents of D35 chickens were chosen for follow-up experiments (37).

The results of this study showed that CY could cause intestinal structure damage, intestinal villi fracture and atrophy, and crypt depth increase in chickens. Surprisingly, the morphology and structure of the ileum and jejunum were intact after CSP treatment, with orderly and slender intestinal villi and shallow crypts, and intestinal injury was improved. CSP increased the intestinal villus height, decreased crypt depth, and enhanced the V/C ratio. In general, CSP can improve intestinal injury, restore intestinal digestion and absorption capacity, and protect intestinal mucosal function. Tight junction proteins are the main connection between intestinal epithelial cells, which play an important role in maintaining the integrity of the intestinal mucosal barrier structure and promoting the normal intestinal mucosal barrier function (38). Studies have shown that polysaccharides can restore CY-induced intestinal mucosal damage by upregulating proteins associated with tight junctions (39, 40). For example, ganoderma lucidum polysaccharide and bovine Dali polysaccharide reversed the damage of intestinal mucosa by upregulating the expression of tight junction proteins (41, 42). Our study confirmed this finding, and CSP played a protective role in cY-induced intestinal mucosal injury by upregulating the expression of Occludin and Zo-1 proteins. In conclusion, CSP is mainly achieved by improving the morphology of the intestinal structure, upregulating the expression of tight junction proteins, and increasing the V/C ratio to intestinal mucosal injury and intestinal damage caused by CY.

Toll-like receptors (TLRs) recognize unique pathogen-associated molecular signatures and are the first line of defense against invading pathogens. TLRs play an important role in immune cell regulation, survival, and proliferation and is widely distributed in intestinal immune cells, playing an important role in the intestinal innate immune system (43, 44). Signal transduction of the TLR pathway starts from the intracellular TIR domain of the receptor and binds to the adaptor protein MyD88, which also contains the TIR domain and further mediates the activation of the NF-κB signaling pathway, thereby inducing and promoting the production of inflammatory factors (45, 46). Studies have found that the immunomodulatory ability of polysaccharides is closely related to that of TLR4 (47). It has been suggested that APS can regulate the host immune system by activating the TLR4-mediated MyD88-dependent signaling pathway (47). The *Solanum nigrum* Linne polysaccharide can regulate immunity by enhancing the expression of key nodes in the TLR4-MyD88 signaling pathway *in vitro* and *in vivo* (48). First, it is noteworthy that CY used in this study can activate the NF-κB signaling pathway, activate the transcription and expression of inflammatory genes, downregulate the expression of tight junction protein, and thus disrupt the intestinal structure and function (49, 50). The results showed that CY induced downregulation of TLR4, MyD88, and NF-κB, while CSP reversed this phenomenon and promoted upregulation of TLR4, MyD88, and NF-KB in intestinal key immune signals. Claudin1 is a major functional protein that tightly binds cells in the intestinal tract, maintaining the balance and stability of the intracellular environment through cell barrier and signal transduction (51). Zo-1 is an important component of tight junction proteins. The downregulation of Zo-1 gene expression will affect the formation of tight junctions between cells, making it difficult for intestinal mucosa to uphold the barrier function (52). In conclusion, CSP can upregulate the expression of *TLR4, MyD88, NF-*κ*B, Claudin1*, and *Zo-1* genes. CSP regulates the intestinal immune system and protects the intestinal mucosal barrier function by activating the TLR4/NF-κB-mediated signaling pathway and upregulating tight junction proteins.

Studies have shown that the microbial flora in the gut interacts with the immune system, which is crucial for host growth and development, immune regulation, and maintenance of intestinal health (16, 33). Disruption of the gut microbiome can lead to an imbalance of the immune system and a compromised gut immune barrier (53, 54). Studies have shown that natural polysaccharides can enhance immunity by continuously stimulating the host immune system, thereby triggering an immune response from the microflora (18). For example, cultured *Cordyceps sinensis* polysaccharides can increase the abundance of probiotics and reduce the production of pathogenic bacteria, alleviating the side effects caused by CY (39). Polysaccharides from Yingshan Yunwu tea can reduce the abundance of Bacteroidetes, Lactobacillus, and Proteobacteria, which provide a new reference for poultry breeding (55). First, according to the results of PLS-DA analysis, there was a deviation in the bacterial community structure between groups M and N, which indicated that CY had an impact on the intestinal bacterial community structure, and the CSP we used also had a positive impact on such results. The Venn diagram showed that CY had a negative effect on species abundance, and surprisingly, CSP could improve the decline in species abundance caused by CY and increase OTUS in the bacterial population. Next, we analyzed the intestinal flora of different taxonomic levels, especially phyla and genus. Firmicutes and Bacteroidetes constitute the majority of bacteria, and polysaccharides can be used as their main energy source and can induce a variety of glucosidase activities of Bacteroidetes (56). Our study found that the relative abundance of Firmicutes in group M decreased, so we inferred that the immunosuppressive effect of CY on chickens might be related to Firmicutes (57, 58). However, at the phylum level, there was no significant difference in the expression of these four groups, which may be due to the disturbance of intestinal flora caused by the immunosuppressive state in the early stage of chicken, which was alleviated to some extent by its immune regulation after CY lost its effect. At the genus level, we found that CSP produces large amounts of Lactobacillus, responsible for the large amount of lactic acid produced by fermenting sugar, which is beneficial bacteria that aid digestion and maintain gut health (59–61). In conclusion, CSP maintains the intestinal microflora and improves the intestinal environment by increasing the species abundance in the intestinal microflora and regulating the bacterial types at the phylum level. CSP improved the intestinal flora imbalance caused by CY and played a positive role in the recovery of intestinal immunity and digestive ability. LEfSe analysis showed that the scores of Lachnoclostridium, Lachnospiraceae_NK4A136_group, Pseudoflavonifractor, and Lactobacillus_gasseri_K7 in the CHM group were higher, suggesting that Lactobacillales are the key system type that CSP acts on intestinal flora, while Lactobacilli belong to Firmicutes. This suggests that CSP can produce acetic acid, lactic acid, and antibacterial substances by treating Firmicutes to prevent pathogens from interfering with health. However, there are also some pathogenic bacteria in Firmicutes that should not be ignored (60, 61). In summary, CSP can regulate intestinal key systems and the structure and composition of microbial communities. The results of this study confirmed that CSP, as a natural plant polysaccharide, can not only improve the diversity of the cecum microbial community but also promote the production of beneficial bacteria and has the superior potential to regulate intestinal microbes.

## Conclusion

To sum up, CSP can restore the BW, protect immune organs, encourage the secretion of cytokines and immunoglobulins in serum, and regulate body's humoral and cellular immunity. CSP can repair damaged intestinal mucosa, improve the expression of tight junction proteins, upregulate the expression of *Claudin1 and Zo-1* genes, and activate intestinal immune-related genes *TLR4, MyD88*, and *NF-KB*, thereby activating TLRs and the NF-κB signaling pathway, regulating intestinal immunity, and protecting the intestinal mucosal barrier function. In addition, CSP can increase the diversity of the microbial community, regulate the structure and composition of the microbial community, and restore the intestinal microbial imbalance. In general, CSP can enhance intestinal health and improve the host's systemic immune response by regulating the intestinal immunity. These results present data-driven support for the development of CSP-based immune enhancers. However, the deeper mechanism of CSP regulation of intestinal immunity is yet to be explored.

## Data Availability Statement

The datasets presented in this study can be found in online repositories. The names of the repository/repositories and accession number(s) can be found in the article/Supplementary Material.

## Ethics Statement

The animal study was reviewed and approved by Guangxi University.

## Author Contributions

YC, WS, JL, and HS conceived and designed the entire trial. YC, YuW, FL, XC, XP, and YiW conducted animal experiments. YC analyzed the data and drafted the manuscript. YC, WS, QL, and KW revised the manuscript. JL and HS directed the whole experiment. All authors have read and approved the final manuscript.

## Funding

The Key Research and Development Plan of Guangxi, China (AB19245037), the Natural National Science Foundation of China (317607446), the Major R&D Project of Nanning Qingxiu District (2020005), and the Major R&D Project of Nanning (20212138) financially supported this work.

## Conflict of Interest

The authors declare that the research was conducted in the absence of any commercial or financial relationships that could be construed as a potential conflict of interest.

## Publisher's Note

All claims expressed in this article are solely those of the authors and do not necessarily represent those of their affiliated organizations, or those of the publisher, the editors and the reviewers. Any product that may be evaluated in this article, or claim that may be made by its manufacturer, is not guaranteed or endorsed by the publisher.
