# Supplementary material for: Effects of Caulis Spatholobi Polysaccharide on Immunity, Intestinal Mucosal Barrier Function, and Intestinal Microbiota in Cyclophosphamide-Induced Immunosuppressive Chickens
Source: Front Vet Sci. 2022 Mar 18;9:833842. doi: 10.3389/fvets.2022.833842 (PMC8972122; doi:10.3389/fvets.2022.833842)
Supplement: Supplementary file 1 [file Data_Sheet_1.docx]

**Supplementary Figures S1.**Rarefaction curve, Shannon-Wiener curve, Rank-productivity curve and Species Accumulation curve results**
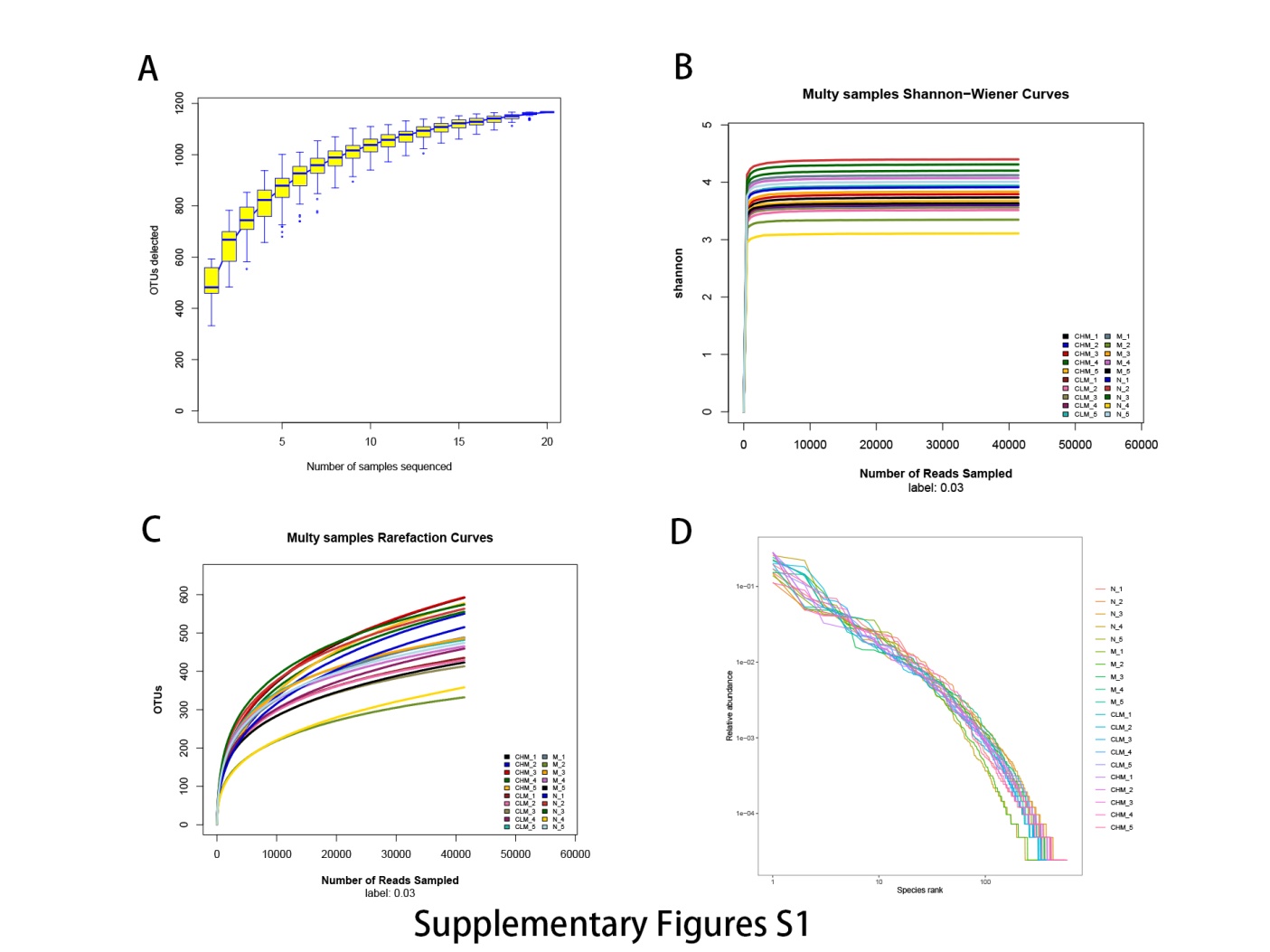
**
