# Supplementary material for: Effects of Caulis Spatholobi Polysaccharide on Immunity, Intestinal Mucosal Barrier Function, and Intestinal Microbiota in Cyclophosphamide-Induced Immunosuppressive Chickens
Source: Front Vet Sci. 2022 Mar 18;9:833842. doi: 10.3389/fvets.2022.833842 (PMC8972122; doi:10.3389/fvets.2022.833842)
Supplement: Supplementary file 2 [file Table_1.docx]

**Supplementary Table 1. Primers used in Quantitative Real-time PCR**

| **Target gene** | **Primer sequence(5’-3’)** |
| --- | --- |
| **Tlr4** | \| GTTTGACATTGCTCGGTCCT \| \| --- \| |
|  | GCTGCCTCCAGAAGATATGC |
| **MyD88** | CCGTATGGGCATGGAACAGA |
|  | CTGGCAAGACATCCCGATCA |
| **NF-KB** | TTGCTGCTGGAGTTGATGTC |
|  | TATGTGAAGAGGCGTTGTGG |
| **Claudin1** | CATACTCCTGGGTCTGGTTGGT |
|  | GACAGCCATCCGCATCTTCT |
| **Zo-1** | TATGAAGATCGTGCGCCTCC |
|  | GAGGTCTGCCATCGTAGCTC |
